# Enhancing the Accuracy of Non-Invasive Glucose Sensing in Aqueous Solutions Using Combined Millimeter Wave and Near Infrared Transmission

**DOI:** 10.3390/s21093275

**Published:** 2021-05-10

**Authors:** Helena Cano-Garcia, Rohit Kshirsagar, Roberto Pricci, Ahmed Teyeb, Fergus O’Brien, Shimul Saha, Panagiotis Kosmas, Efthymios Kallos

**Affiliations:** 1 Medical Wireless Sensing Ltd., London E1 2AX, UK; roberto.pricci@metamaterial.com (R.P.); fergus.obrien@metamaterial.com (F.O.); shimul.saha@metamaterial.com (S.S.); themos.kallos@metamaterial.com (E.K.); 2 Metamaterial Inc., Dartmouth, NS B2Y 4M9, Canada; 3 Brunel Innovation Centre, Cambridge CB21 6AL, UK; rohit.kshirsagar@brunel.ac.uk (R.K.); ahmed.teyeb@brunel.ac.uk (A.T.); 4 Faculty of Natural and Mathematical Sciences, King’s College London, Strand, London WC2R 2LS, UK; panagiotis.kosmas@kcl.ac.uk

**Keywords:** non-invasive, glucose, diabetes, monitor, near infrared, radiofrequency

## Abstract

We reported measurement results relating to non-invasive glucose sensing using a novel multiwavelength approach that combines radio frequency and near infrared signals in transmission through aqueous glucose-loaded solutions. Data were collected simultaneously in the 37–39 GHz and 900–1800 nm electromagnetic bands. We successfully detected changes in the glucose solutions with varying glucose concentrations between 80 and 5000 mg/dl. The measurements showed for the first time that, compared to single modality systems, greater accuracy on glucose level prediction can be achieved when combining transmission data from these distinct electromagnetic bands, boosted by machine learning algorithms.

## 1. Introduction

Diagnostically accurate non-invasive sensing of blood glucose levels is one of the most desired goals in medicine, as diabetes is a growing chronic disease that is affecting almost 1 in 10 adults [1]. While some minimally invasive sensing systems are rapidly gaining commercial traction [2,3], the vast majority of people living with diabetes still rely on painful, invasive methods to manage their condition, based on drawing blood or interstitial fluids to measure their blood glucose concentration. Minimally invasive systems primarily rely on sampling the glucose in the interstitial fluid, either via implantable enzymatic sensors [4,5] or by attempting to transdermally extract the interstitial fluid with techniques such as inverse iontophoresis, ultrasound, and thermal activation [6,7,8]. These minimally invasive techniques could serve as a steppingstone until a fully non-invasive solution becomes clinically available, as long as issues such as accurate correlation with blood glucose and skin irritation are addressed.

A variety of alterative, non-invasive approaches have been investigated in the past 40 years, such as infrared spectroscopy (including absorption and Raman spectroscopy), bioimpedance spectroscopy, and radio wave sensing [9,10,11,12,13,14,15,16,17,18]. It is well known that any such non-invasive technique must overcome significant challenges to obtain clinical sensing accuracy, such as the weak signature of the glucose molecule compared to other tissue components and water, the signal interference from other substances, and the effects of environmental factors such as ambient temperature and skin moisture.

One approach to improve accuracy and specificity involves data processing using artificial intelligence methods, a technique now common in many fields of medicine [19]. Estimations of glucose levels using machine learning (ML) algorithms have been applied with promising results in data obtained from a variety of non-invasive sensors such as photoplethysmography (PPG) [20,21], near infrared [22,23], and microwave [24].

Along different lines, another approach to improve the performance of non-invasive glucose sensors involves the use of multiple sensors and particularly sensors of different modalities. For example, Caduff et al. [25,26] simultaneously collected data from an optical (568–798 nm) and dielectric impedance (up to 1 GHz) system, while Yadav et al. [21] utilized PPG, galvanic skin response, and temperature sensors. Guevara et al. [27] used diffused reflectance (700–1000 nm) and impedance spectroscopy (1–200 MHz). The role of the additional sensors in such multimodal systems can be either to provide independent data on the glucose level changes, and/or to minimize the factors that indirectly affect the glucose sensing such as ambient temperature or patient motion. 

In this work, we combined two independent non-invasive glucose-sensing modalities, near infrared (NIR) absorption spectroscopy, and radio frequency (RF) transmission using millimeter waves, together with machine learning data processing, to improve the detection of glucose level changes in aqueous samples. Each modality thus relies on a different underlying physical mechanism to detect glucose changes. In addition, our multiwavelength system was designed to combine the NIR glucose-sensing data with an RF signal that interrogated the sample in transmission mode. This can provide more valuable RF-sensing data compared to reflection or surface sensors used in the previous multimodal efforts, as it ensures that the signal interacts with the whole sample thickness. Furthermore, in this work we were able to quantify how each sensor modality contributes to the accuracy in glucose prediction, something that has not been available in the earlier multimodal systems.

Due to this distinct nature of the sensing systems, neural networks proved inadequate in predicting glucose concentration levels. Instead, we implemented an ML technique using artificial neural networks for the data processing. Our work shows for the first time that when we collected multiwavelength-sensing data from these two distinct electromagnetic bands, we achieved increased accuracy in predicting the glucose levels, compared to the prediction accuracy of using each sensing system alone.

## 2. Materials and Methods

### 2.1. Hardware and Data Collection

**Figure 1:**
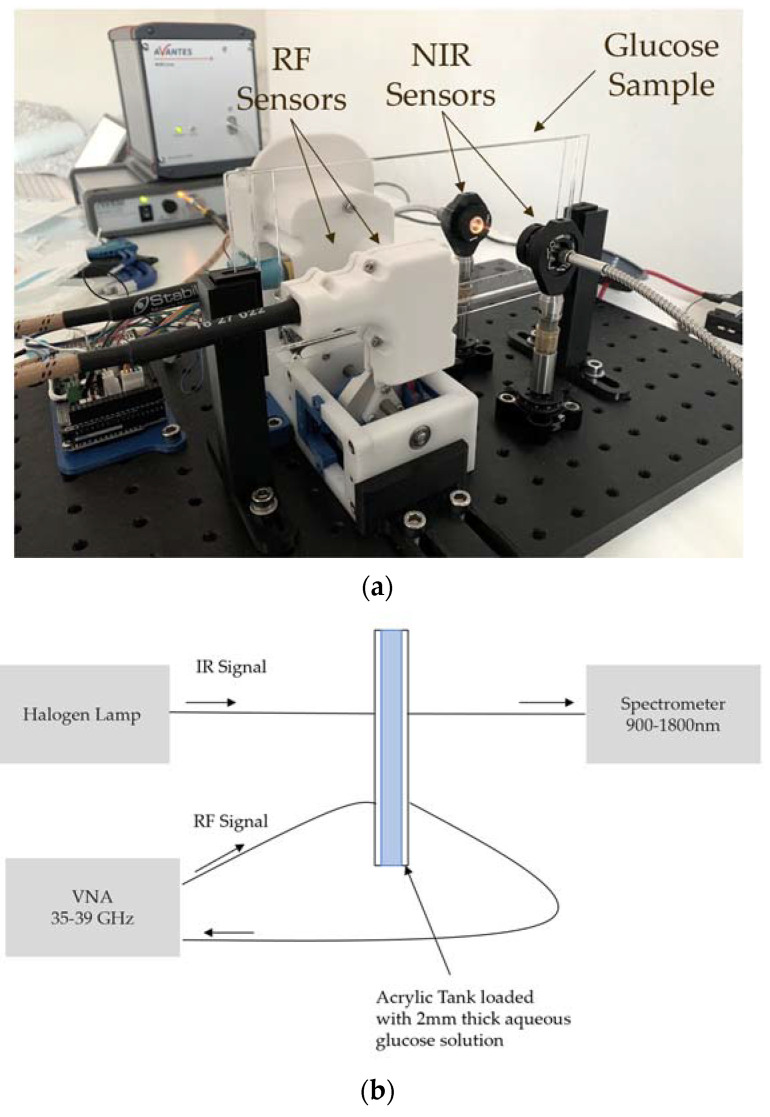
(**a**) Photography of the experimental setup consisting of 2-mm wave sensors enclosed on a custom-made 3D printed case, two optical fibers, and an acrylic tank containing the glucose samples; (**b**) schematic view of the experimental setup.

The multiwavelength system operates on two different electromagnetic bands, RF millimeter waves and NIR. The RF-sensing subsystem is equipped with two opposingly facing patch antennas operating between 37 and 39 GHz [28]. The antennas are placed in a custom-made 3D printed holder with the sample under test sandwiched between them (Figure 1) The antennas are driven by an MS46122B (Anritsu, Kanagawa, Japan) vector network analyzer (VNA) using a pair of Stability Plus cables (MauryMicrowave, Ontario, CA, USA). As detailed in [29], it is beneficial to operate at the maximum possible radio frequency, as this leads to larger changes in the received signal due to the increased electrical length of the sample. This upper frequency, however, is limited by the uncertainty (lower signal-to-noise ratio) introduced by the VNA due the decreased received signal amplitude for higher frequencies. The frequency range 35–40 GHz is within this optimal limit for the given sample thickness [29]. Operating below 40 GHz also has the additional practical advantage that the RF components have lower losses and are more readily available.

The NIR subsystem consists of two aligned optical fibers, also opposingly facing, with the same sample placed in between. One fiber is connected to a broadband halogen lamp (AvaLight-Hal-S from Avantes, Louisville, CO, USA) light source and the other to a spectrometer (NIRLine AvaSpec-NIR256 from , Louisville, CO, USA). Both sensors were thus placed in a transmission configuration (Figure 1b) The NIR wavelength band of 900–1800 nm was chosen as it is well known to (i) feature molecular vibration and rotation of bonds in the glucose molecule, (ii) transmit well through the skin, and (iii) have affordable, widely available hardware components [30].

The samples under test were contained in an acrylic tank with dimensions 170 by 70 by 6 mm. The tank had a pair of 2-mm-thick walls, creating a 2-mm gap for holding the glucose-loaded solutions. The aqueous glucose solutions were made following the same procedure as in the previous work [31], where the samples were mixed with a hand mixer (frapediera) until they appeared to be completely homogenous. During the sample preparation, different amounts of glucose powder (C_6_H_12_O_6_, anhydrous D-(+)-Glucose) were mixed with deionized water to produce samples with the following concentrations: 0, 80, 140, 300, 600, 800, 1100, 1400, 3000, and 5000 mg/dl.

Prior to the measurements, the VNA and the spectrometer were calibrated. The VNA was calibrated performing a short-open-load-thru (SOLT) calibration, while the spectrometer was calibrated using the transmission through the empty tank as a reference and the signal received when the bulb was off as dark. 

Multiple rounds of measurements were performed. A single measurement round consisted of measuring RF and NIR data for all ten glucose samples. The measurements using the RF sensor and the NIR sensor were taken sequentially in a span of a few seconds. Additionally, the sample temperature was measured for each sample. Between swapping different samples, the tank was rinsed with deionized water and then dried using a paper towel, to avoid cross-contamination. To ensure repeatability on the obtained results, a total of 17 rounds of measurements were performed. From these 170 measurements, 36 measurements were discarded due to experimental errors, yielding a dataset with a total of 134 useful data points. 

### 2.2. Glucose Concentration Prediction

Once the measured data were collected, different computational models were used to predict the glucose concertation. It was evident from preliminary processing that the correlation between the combined inputs to the models (RF and NIR) and the output (glucose concertation) was nonlinear. Such high nonlinearity could not be incorporated in regression models. Consequently, machine learning models were considered. One of the most commonly used models for such prediction is based on artificial neural networks (ANNs).

For the development of ANN, the entire dataset acquired through experiments was divided into a training set and a test set. A training set typically consisted of 80% of the data while the generalization of the models was tested using the remaining 20%. The steps for the development of the ANN included: (i) Segregation of training and test datasets; (ii) selection of ANN architecture using k-fold cross validation; (iii) selection of training termination criteria and training; (iv) validation of the computational models using the test dataset.

The ANNs in this study were developed in Python 3.6 programming language using the Tensorflow 2.3 release (Google Brain, Mountain View, CA, USA). Out of the 134 data points available from the experiments, 114 were used for training the ANN while 20 were reserved for testing. For the selection of the ANN architecture (number of hidden layers and the number of neurons in every layer), a method known as k-fold cross validation was used. In this method, the training dataset was further divided into a number of smaller datasets. Each of these are turn-wise reserved for testing, while an ANN is training using the other datasets. In this case the data was divided into 5 smaller sets. Different network architectures were trialed and the best architecture containing a single hidden layer with 64 neurons was chosen for the model development. 

Three different ANN models were eventually developed, each using a different number and type of inputs. The first model used only the data from the RF sensor for the prediction of glucose content. The second model used only the NIR data, while the third model combined the NIR and the RF data as the input. In order to ensure that all the models were trained on the same total number of data points, the third model only used half the number of total training points initially reserved for training. Consequently, it was trained using 67 trials, each containing 10 inputs. The individual RF and NIR models were trained using 134 trials, each containing 5 inputs. This made the total number of points used for training 670 for all models. 

The RF and NIR multiwavelength hardware used in this work provided multiple data inputs, such as signal amplitude for a very large number of wavelengths, RF reflection amplitude data, and RF phase data. In order to be able to build future multiwavelength systems with as simplified hardware as possible, we restricted the input data used in these models to 1 RF wavelength and 3 NIR wavelengths. For all the models the temperature of the sample was also considered as an input since it is known to significantly influence the readings.

It is critical that the computational models are not overfitted (test error higher than training error). For achieve this, the termination criteria for the training must be carefully chosen. In this study, the models were trained for a fixed number of iterations, carefully monitoring the training and the test error. When the average test error was observed to go beyond the training error, the training was immediately terminated. Additionally, the training was also terminated if there was no significant improvement observed in the error on continued iterations. 

To evaluate the predictive accuracy of the different machine learning models, the mean absolute relative difference (MARD) metric was used [12,32]. MARD is one of the most common metrics used to assess the performance of continuous glucose monitoring systems. MARD calculates the average of the absolute error against a reference value:

(1) $$MARD=\frac{1}{N}\sum_{i=1}^{N}\frac{\left|y_{ri}-{\hat{y}}_{i}\right|}{y_{ri}}\times 100,$$

where *𝑦_r_* is the reference glucose levels for the *N* sample points and $\hat{y}$ represents the estimated glucose levels provided by the machine learning model. The smaller the *MARD* value is the more accurate the system is.

## 3. Results

**Figure 2:**
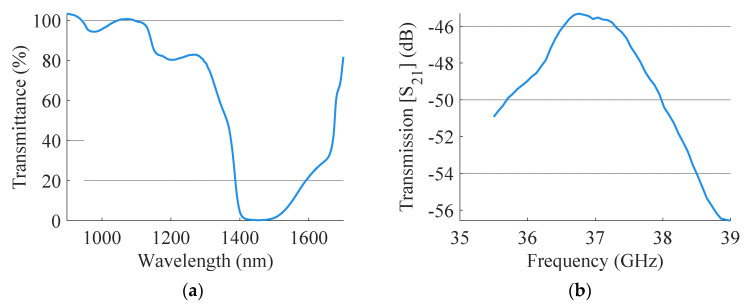
(**a**) NIR transmittance for a water sample (0 mg/dl); (**b**) RF transmission amplitude for a water sample (0 mg/dl).

The setup of Figure 1 was used to measure transmission and reflection amplitude for the RF sensors and transmittance for the NIR sensors. The raw data for one of the measurement rounds is presented in Figure 2.

Each round of measurements was slightly different due to small differences in sensor placement, antennas used (since these were replaced between the measurement rounds), and ambient temperatures. To remove non-glucose related effects, the 0 mg/dl measurement for each round was taken as a reference value for that specific round.

**Figure 3:**
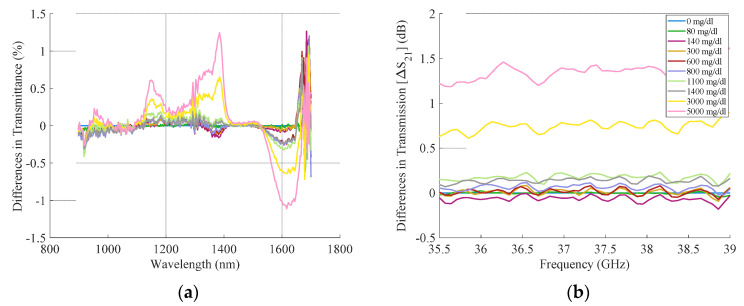
(**a**) Difference in NIR transmittance for a measurement round using 0 mg/dl as a reference measurement (glucose concentrations between 0 to 5000 mg/dl); (**b**) difference in RF transmission for one measurement round using 0 mg/dl as a reference (glucose concentrations between 0 to 5000 mg/dl).

Figure 3 shows the raw data of one measurement round when using the 0 mg/dl sample as a reference for the NIR (Figure 3a) and the RF sensors (Figure 3b). The NIR data (Figure 3a), when referencing to 0 mg/dl, provided information about how the amplitude at different wavelengths responds to changes in glucose. These data indicated that wavelengths around 1390 and 1620 nm are more sensitive to detecting glucose changes. From these two wavelengths, further processing was performed with the data acquired for 1390 nm since it presented smaller standard deviation between measurement rounds than 1620 nm (0.064% for 1390 nm and 0.135% for 1620 nm). Regarding the RF data (Figure 3b), all the frequencies showed similar behavior, as there was no known glucose-specific resonance in this part of the spectrum. To choose the best RF frequency for the ML processing, we considered the antenna performance: We evaluated the reflection amplitude S_11_ of the antennas to remain under −10 dB, and additionally minimize the standard deviation on the transmitted signal for each round. Although this optimal frequency was not exactly the same for all the measurement rounds, it was always close to 36.5 GHz. Therefore, we chose 36.5 GHz as the main RF frequency for all rounds.

**Figure 4:**
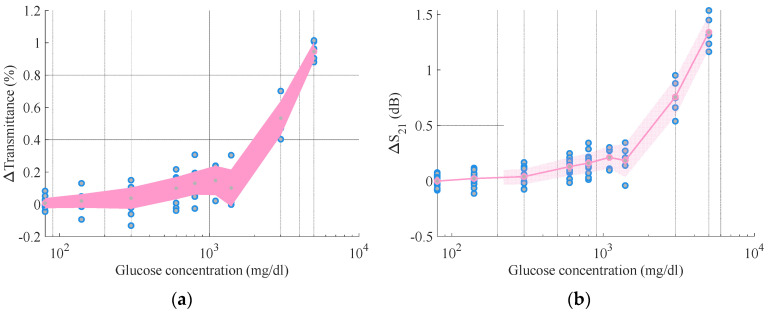
(**a**) Mean of the NIR transmittance at 1390 nm for all the measurement rounds for different glucose concentration values; (**b**) mean of the RF transmission amplitude (S_21_) at 36.5 GHz for all the measurement rounds and different glucose concentration values. For (**a**,**b**), the blue dots represent the datapoints and the pink trace represents the mean of all these points. The pink shaded area represents the measurement error obtained by calculating the standard deviation of the measurements. Note the X axes have logarithmic scale.

To show the relationship between the sensor data and the glucose amount, the mean of all the rounds was plotted including the standard deviation as error (Figure 4).

In line with previous work by the authors [29,31,33], a linear relationship with a sensitivity of 0.00026 dB/(mg/dl) was observed for the RF mm-wave data, Figure 1b, between the transmission S_21_ and the amount of glucose concentration in the samples. This sensitivity is comparable to the sensitivity of other transmission-based RF systems at lower frequencies, which can range between 0.00008–0.00140 dB/(mg/dl) [29,34,35,36,37]. This approximately linear relationship is also evident for the NIR transmittance data, with a sensitivity of 0.00019%/(mg/dl) (Figure 1a). Note that the data in this figure are plotted against a logarithmic axis.

Next, we wished to perform predictions of glucose levels and study how data of two different types of sensors impacted the overall system sensitivity. To achieve that, all the raw data were fitted on a machine learning algorithm. Three different machine learning models were trialed to find the most suitable for the prediction of glucose content in the samples: RF data only, NIR data only, and combined RF + NIR data. The objective was to examine if and by how much the glucose predictions were improved when using datasets that combine readings from both RF and NIR data, compared to using data from one modality only.

**Figure 5:**
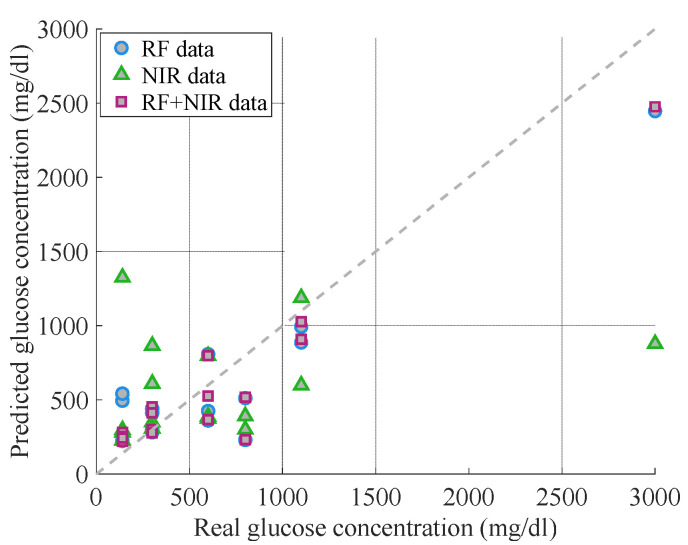
Predicted glucose concentration obtained after applying three ML models against the real glucose concentration. The circle datapoints represent the predicted values obtained when using the ML model tailored for the RF data only (MARD: 76%); the triangle datapoints represent the predictions when applying an ML model only to the NIR data (MARD: 127%); and the square datapoints represent the predicted values when using a ML model using RF and NIR data combined (MARD: 46%). The dashed line indicates the loci of the points where the reference and predicted values would match.

Figure 5 presents the predicted values against the real glucose values for the three different machine learning models used. We observed that, for most data points, the predictions of the model using RF dataset were closer to the reference values of glucose concentrations. The predictions using the NIR data appeared poorer, while the predictions of using combined datasets appear improved. The model also significantly underestimated the glucose concentrations when extremely high values (3000 mg/dl) were used.

To quantitively compare the performance of the three machine learning models, we calculated the MARD values including only the clinically relevant glucose concentrations (140–800 mg/dl). The calculations showed that the prediction using the NIR-only dataset had a MARD value of 127%, the prediction based on RF-only data had a value of 76%, and when combining both methods, the MARD value was further reduced to 46%. The combined model thus shows the greatest accuracy in predicting glucose levels, compared to using datasets from a single modality.

## 4. Discussion

### 4.1. Multiwavelength-Sensing Analysis

To interpret the results of the multiwavelength sensing, we needed to consider the distinct physical mechanisms of signal variation (RF or NIR) due to glucose concentration changes.

For the NIR band, the light is primarily attenuated due to electronic absorption of the NIR photons. The measured attenuation of light follows, to a first approximation, the Beer-Lambert law [38]. In more complex samples, such as blood or human flesh, the light would be primarily diffusively scattered, and not all photons would travel the same path distances through the sample. These distances can thus be much greater than the sample thickness [38]. However, these effects are not strongly present in the weakly scattering water-based samples used in this study.

For the RF band, signal attenuation is primarily caused by the differences in absorption due to molecular rotation of the water and glucose molecules, manifested via the imaginary part of the sample permittivity [29,33]. While there is no resonant absorption signature for glucose in the RF band, the RF attenuation is excellent at detecting changes in the water concentration, which is a parameter strongly affecting the NIR signal absorption. This is a possible key reason why the prediction accuracy increases when the NIR data are assisted by the RF data. 

Thus, there are two independent mechanisms of glucose detection, electronic absorption and dielectric absorption, for the NIR and RF signals, respectively. The MARD values are different depending on which data are used to estimate the glucose concentration, which is a combination of the physical mechanism and the experimental apparatus used for each band.

There are additional factors that may affect the MARD values. The RF method captures a larger section of the sample and therefore the measurement is an average of a bigger sample size. In addition, the NIR signal has a much shorter skin depth, and is primarily more sensitive to the sample portion near the surface. The RF signal has a longer skin depth and interacts more strongly with a deeper section of the sample, compared to the NIR signal. In more complex biological samples, we expect superficial skin features to influence the NIR, while the RF signal can provide information on the bulk of the sample.

### 4.2. Comparison with Other Techniques

It is useful to compare the sensing errors and sensitivity of the multiwavelength system presented in this work to other approaches, keeping in mind that the current system was designed around combining modalities with the goal to boost the accuracy of individual glucose-sensing modalities. 

Invasive glucose meters designed for diabetes management typically must satisfy the accuracy standard of ISO15197:2013, which requires less than ±15% error for concentrations higher than 100 mg/dl, for at least 95% of all measurements. An accuracy of ±15 mg/dl is required for smaller values [39]. Clinically approved minimally invasive systems can offer aggregate MARD values around 11%, with approximately half of all readings being within 10% of a reference capillary blood glucose value [5]. Non-invasive efforts, such as impedance, photoacoustic, NIR diffuse reflectance, and optical absorption, have shown MARD values of 5–8% [40]. In terms of multi-modal systems, the non-invasive optical and dielectric impedance systems reported by Caduff et al. and Guevara et al. [27] achieved a MARD of 27% and root-mean-square error of 22 mg/dl in human subjects, respectively [25]. It should be noted that these relatively small MARD values reported for non-invasive systems are usually for limited number of subjects in well-controlled studies. These numbers worsen when the number and type of participants is enlarged and are performed under real world conditions [9].

The MARD values obtained in this work, down to 46%, are clearly not adequate yet to satisfy clinical glucose-sensing requirements. However, the aim of our work has been primarily to demonstrate the glucose prediction improvements by using the novel approach of multiwavelength sensing, and secondarily to maximize the absolute sensing accuracy. We expect that further absolute MARD reduction can be achieved by combining the techniques presented in this work with more sophisticated NIR hardware that has been presented in the literature [40]. As an example, NIR systems in the same wavelength range examined here, unassisted by RF sensing, reported accuracy errors on the range of ±15–50 mg/dl [41,42,43,44]. Adding mm-wave RF sensing to equivalent systems could be enough to bring them within a clinical accuracy range, since this is the first multi-modal system that utilizes transmission of radio waves to enhance sensing accuracy.

To improve the sensing accuracy of our system, especially at lower glucose concentrations, larger signal-to-noise ratios need to be achieved using more sophisticated optical and RF systems. For example, high-power single-wavelength laser diodes covering different discrete wavelengths can be included, along with a sensitive photodetector diode. A suitable diode cooling system should be used to ensure wavelength and output power stability. The RF system can benefit by using dedicated narrowband signal generators and receivers, rather than a VNA. Lock-in amplifiers and signal modulation also provide improved precision. Finally, the accuracy is expected to improve as more data are collected and the ML models are better trained. These approaches are well known in the literature and some of them will be incorporated in future iterations of the multiwavelength-sensing method presented in this work. 

## 5. Conclusions

In this paper, we presented a multiwavelength glucose-sensing system that combines RF mm-wave and NIR signals to provide more accurate glucose readings on controlled samples. The system consisted of two patch antennas operating at 36.5 GHz and two optical fibers carrying light between 900–1800 nm, all of them simultaneously placed on opposing sides of a sample. The experimental measurements were performed on aqueous glucose solutions with concentrations ranging between 80 mg/dl and 5000 mg/dl. We showed using machine learning modeling that higher accuracy in the prediction of the glucose concentration was obtained when data from both modalities were used.

Optical radiation is commonly used for the non-invasive sensing of biological parameters such as oxygen saturation, heart rate, or respiration rate. However, it is difficult to obtain clinically relevant accuracy for body analytes and blood constituents such as glucose, especially in compact form factors such as bracelets or smartwatches. The work reported here shows that it is beneficial to consider collecting readings from a different modality, such as RF, as it can improve the accuracy of sensing. The mm-wave part of the spectrum is particularly interesting due to the small form factor of its components. While the work presented uses a relatively simple optical-sensing system, the concept of combining different parts of the electromagnetic spectrum (multiwavelength sensing) could be applied to improve the sensing accuracy of more sophisticated, commercially mature systems targeted at medical sensing.

## 6. Patents

Patent applications GB21028808 and GB21028915 relate to work presented in this paper.

## Data Availability

Not applicable.
